# Similarities and differences of a selection of key accreditation standards between chiropractic councils on education: a systematic review

**DOI:** 10.1186/s12998-016-0127-6

**Published:** 2016-12-07

**Authors:** Stanley I. Innes, Charlotte Leboeuf-Yde, Bruce F. Walker

**Affiliations:** 1 School of Health Professions, Murdoch University, Murdoch, Australia; 2 Institut Franco-Européen de Chiropraxie, Ivry sur Seine, France; 3 Complexité, Innovation et Activités Motrices et Sportives, UFR STAPS, Université Paris Sud-11, Orsay Cedex, France

**Keywords:** Councils on chiropractic education, Standards of accreditation, Similarities, Differences

## Abstract

**Background:**

Councils of Chiropractic Education (CCE) indirectly influence patient care and safety through their role of ensuring the standards of training delivered by chiropractic educational institutions. This is achieved by a process of accreditation where CCEs define and assess graduate competencies and educational standards. A previous study comparing CCE graduate competencies found variations between the CCE jurisdictions. It was proffered that variations in standards may potentially compromise patient care and safety and also inter-jurisdictional mutual recognition. This study continues the examination of CCEs by looking for similarities and differences in CCE accreditation standards.

There were two purposes of this review. The first was to compare the accreditation standards, domains of accreditation standards, and components of the domains of accreditation standards as represented by the domains of “Mission, goals, vision, objectives”, “Resources”, “Faculty/Academic staff”, “Educational program/curriculum”. In addition, we compared the accreditation standards between CCEs and those of the widely accepted medical accreditation standards of the World Federation of Medical Education (WFME), in order to search for deficiencies and opportunities for improvements in these standards.

The second purpose was to make recommendations, if significant deficiencies or variations were found.

**Method:**

We undertook a systematic review of the similarities and differences between five CCEs’ definitions of an accreditation standard and the descriptive lists of accreditation standards they have adopted. CCE selection criteria and data selection method were undertaken in a systematic manner. This information was tabulated for a comparative analysis and took place in April 2016.

**Results:**

Only two CCEs had a definition of the term “accreditation / educational standard”. At the domain level there was considerably more similarities than differences. The differences became more apparent when the comparisons were made at the component level. These included intended purposes of the mission statement, standards for faculty staff, requirements for clinical training by students, program budgetary autonomy and transparency, the inclusion of chiropractic philosophy and history, and which subjects should be taught in basic, behavioural and clinical sciences.

**Conclusions:**

A series of recommendations were made. These included the need for an increased clarity of the required basic and clinical science subjects, teaching clinic student requirements, and faculty staff qualifications. These are proposed with the intention of creating uniform and high quality international accreditation standards for chiropractic education. Future research should compare the levels of CCEs inspection standards and processes to see if similarities and differences exist also there.

**Trial registration:**

Not applicable.

## Introduction

### Background

Worldwide there are many chiropractic programs. Accreditation authorities assess these programs to ensure that certain professional standards are met in chiropractic pre-professional training so that patients receive the best care possible from graduates of those programs.

Accreditation of higher education is a process by which official accrediting bodies evaluate institutions using a set of standards and procedures to assess the contents and level of quality of education provided in the hope of producing competent graduates in specific professions [1]. Obviously, the value of the accreditation process depends on the standards and procedures that form their basis. The use of competencies, policies, accreditation standards and site inspections are now widely recognised as an important basis needed to be able to assess programs for accreditation [2, 3]. Additional objectives of the accreditation process are to ensure quality institutional functioning, to strengthen the capabilities of educational institutions and to provide public confidence in health practitioner educational institutions generally [1]. Within chiropractic, Councils on Chiropractic Education (CCE) are responsible for the accreditation of institutions.

CCEs expect chiropractic programs to train students to attain satisfactory levels of knowledge, skills and attitudes before graduating. These are known as competencies and are specified by CCEs. Previous research has shown that while there are similarities, there is also considerable variation among the CCEs’ written documentation, including definitions of important terms, for entry-level graduate competencies [4]. These definitions and construction of competencies have important implications for the way that competence based medical education is implemented [2]. However, this aspect is only part of the accrediting process. CCEs prescribe a set of educational standards for accreditation of chiropractic programs. These accreditation standards detail, amongst other things, the required program content, facilities, faculty and financial management.

### The problem

There are studies in medical education exploring the impact of accreditation standards prescribed by regulatory or licensing agencies [5–10]. These studies have resulted in a dialogue from which medical programs and regulatory bodies have been able to explore and improve the strengths and weaknesses of the accreditation process. However, there are none in chiropractic education. It is obvious that relevant and uniform standards are needed to ensure patient safety and protection as well as international transportability of professionals [11]. Nevertheless, past research has shown that there are significant variations between CCEs in relation to competencies [4]. This may result in differing requirements and processes of accreditation between these CCEs. If variations also exist in the educational standards of CCEs this may result in differing quality levels of practitioner profiles, which could create differences in the quality of care and patient safety. While there may be several ways of achieving the same high quality graduate attributes, variations of approach may also produce differing levels of quality. Ultimately, an unequal and deficient standard may also impact on the international mobility of chiropractors.

### A comparison standard

In 2004 the World Health Organisation and the World Medical Association approved the World Federation for Medical Education (WFME) project for an international collaboration programme for the reorientation of medical education [12]. This project was concerned with the education and training of medical doctors in order to improve the health of all people through the promotion of high quality medical education [13]. Consequently, the WFME published a set of international standards intended to be used as a tool for quality assurance and development of basic medical education as well as for the evaluation and recognition of accrediting agencies [14]. The most recent revision was published in 2015 [15]. These standards have been used in over 70 countries and over 500 medical schools have now adopted them for their mandatory self-evaluation studies [12]. It is recognized that chiropractic standards will differ in some areas to medicine. However, they both share common basic sciences and clinical sciences which mean that this extensively researched and widely adopted set of standards offers a useful benchmark for an investigation into the similarities, differences and possible deficiencies between chiropractic accrediting agencies.

### Aim

The aim of this systematic review was to answer the question; are there similarities and differences between the various CCEs on the accreditation standards they have adopted? Further, by comparing these to the standards of the WFME and see if there are opportunities for improvement.

### Objectives

The specific objectives were to review and compare the different CCE definitions of:

1. Accreditation standards;
2. Domains of accreditation standards;
3. Components of the domains of accreditation standards as represented by the domains of “Mission, goals, vision, objectives”, “Resources”, “Faculty/Academic staff”, “Educational program/curriculum”.

In addition, we compared the accreditation standards between CCEs and those of the WFME, in order to search for deficiencies and opportunities for improvements in these standards.

## Method

We used the same design as in our previous study, namely a systematic review to investigate the first three objectives [4]. Protocols for clinical systematic reviews are recommended to be prospectively registered (PRISMA [16]). However, as this systematic review focussed on the descriptive definitions in accreditation standards documents and not peer reviewed journal articles, it was not suitable for prospective registration with databases such as PROSPERO [17]. This study, which took place in April 2016, was an analysis of freely available website content and did not involve collecting data from human participants, hence ethics approval was not required.

### Eligibility criteria

The World Health Organisation recommends the Council on Chiropractic Education International (CCE-International) as the source of information regarding evaluation of chiropractic education [18]. Consequently, for CCE inclusion, we used this recommendation meaning that a CCE included in our study had to be recognized by and be a member in good standing of the CCE-Int. At the time of data collection (November, 2015) all the CCEs known to us, i.e. the Council on Chiropractic Education (CCE-USA) [19], Council on Chiropractic Education Australasia (CCE-Australasia) [20], European Council on Chiropractic Education (CCE-Europe) [21], and Council on Chiropractic Education Canada (CCE-Canada) [22], met the inclusion criteria. The CCE-International standards were also included in the analysis [23]. Its function is not, strictly speaking, the same as that of the other CCEs, in that it does not actively inspect chiropractic institution, however it functions as an “umbrella” organisation for all the included CCEs and thus warrants inclusion.

### Data extraction process and synthesis of results

The respective CCE websites were identified and searched independently by the lead author and a research assistant. All CCEs were asked in writing whether additional relevant information was available that was not available on their respective websites.

A Masters in Business Administration graduate experienced with organisational evaluation acted as a research assistant and was instructed on the search domains. A training exercise was undertaken to establish a consistent process for extracting data from the websites. The research assistant was instructed on the aims and objectives of the project. Further, the roles of the CCEs were defined. The lead author and the research assistant then independently searched the CCE websites to identify and extract a definition of an accreditation or educational standard. The extracted data were recorded and tabulated. The author and research assistant then compared these for agreement. A third investigator was available to resolve any conflicts.

The same process was repeated for the extraction of the Accreditation Standards lists for each CCE.

The table format for the definitions was structured to identify similarities and differences with respect to their definitions and descriptions of the concept of “educational standards”, and the four domains of: 1. Mission, vision, goals, objectives, 2. Faculty/Academic staff, 3. Resources, and 4. Educational program/Curriculum.

**Fig. 1:**
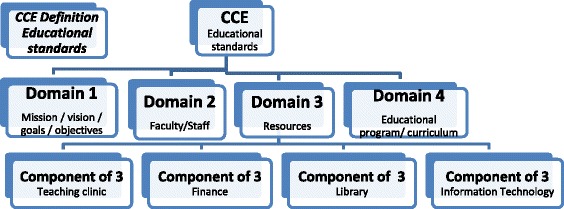
Diagram of the systematic review structure

Finally, the components of the four selected domains were extracted and tabulated, as described in Fig. 1, and thereafter analysed for similarities and differences.

## Results

The research assistant and lead author (SI) agreed on all blindly extracted definitions of an educational standard. There was also agreement on all 5 of the CCE lists of accreditation standards.

### Objective 1: definitions of educational standards

Two definitions of the term “educational standard” were found in the accreditation standards of the five CCEs (Table 1). The CCE-Australasia, which uses the term educational standard instead of accreditation standard, defined it as a criterion used as a model or pattern. The CCE-Europe definition was more extensive and defined it as a set of pre-determined criteria to certify that an institution is providing an education so that its graduates achieve their core competencies. No other definitions were found.

**Table 1:** Definitions of educational standards used by the major regulatory bodies

Name of CCE	Definition of “educational standard”
CCE-Aust	Offers a rule or basis of comparison established in measuring or judging capacity, quantity, quality, content and value; criterion used as a model or pattern. *Pg 20, 2009, Educational Standards for First Professional Awards Program in Chiropractic. CCE-Australasia* [20]
CCE-Canada	No definition found [22],
ECC-Europe	Set of pre-determined criteria by which judgements and/or decisions are made to certify that an institution is providing an education and training to ensure that all its graduates achieve the core competencies. *Pg 62, ECCE Standards, 2013.*[21]
CCE-Int	No definition found [23]
CCE -USA	No definition found [19]
WFME	No definition found [15]

### Objective 2: domains of educational standards

The analysis revealed 13 domains among the five CCEs accreditation standards and nine domains in the WFME standards (Table 2). There were considerably more similarities than differences. The CCE-USA alone had a domain called “Ethics & integrity”. This topic was found as a subdomain of CCE-Australasia and CCE-Europe standards. The CCE-USA also had two unique domains called “Distance/Correspondence education” and “Service”. Since they were single occurrences at the domain level in only one CCE they could not be compared and consequently were not included. This left 10 domains that were common to all CCE standards. Of these 10, a comparison of the domain of “Research/Scholarship” has been presented elsewhere [24]. Further the domains of “Program evaluation”, “Continuous renewal/Improvement”, “Student services” and “Student admissions” will be the subject of a future study and were excluded.

**Table 2:** Comparison of Educational standards Domains of CCEs

Major Elements/Domains of Educational standards	WFME	CCE-Aust	CCE- Can	ECC-Europe	CCE-Int	CCE-USA
Mission/Planning/Assessment/Vision/Goals/Objectives	X	X	X	X	X	X
Resources/Educational resources	X	X	X	X	X	X
Educational program/Curriculum	X	X	X	X	X	X
Faculty	X	X	X	X	X	X
Scholarship/Research and relationship to teaching		X	X	X	X	X
Student admissions	X	X	X	X		X
Student services	X	X	X	X		X
Governance/Administration/Structure/Organisational factors	X	X	X	X	X	X
Ethics & integrity						X
Continuous renewal & improvement	X	X	X	X	X	X
Distance/correspondence education						X
Service						X

For the purposes of this review we concentrated on the remaining four important domains of;

1. Mission/Vision/Goals/Objectives
2. Faculty/Staff
3. Resources
4. Educational program/ Curriculum

### Objective 3: comparative analysis of four domains of educational standards

#### Domain 1 of accreditation standards: mission/vision/goals /objectives

All CCEs documentation included the requirement for educational programs to clearly define their mission/vision/goals/aims/objectives (Table 3). The CCE-Australasia expected the mission statement to be based on input by its principal stakeholders. Principal stakeholders were listed as staff, students, community, education and health care authorities, professional organizations and post-graduate educators. The CCE-Europe and CCE-USA made it a requirement to make the mission statement known to all stakeholders.

**Table 3:** Descriptions used by CCEs of the three selected representative domains of “Mission, goals, vision, objectives”, “Resources”, “Faculty/Academic staff”

Educational Standard : Mission/goals/vision/objectives	WFME	CCE-Aust	CCE-Can	ECC-Euro	CCE-Int	CCE-USA
Provide/define a mission/goal/objective statement	X	X	X	X	X	X
Based on input by stakeholders		X				
Made known/available to all stakeholders	X	X		X		X
Describe the desired graduate as competent primary contact practitioner	X	X	X	X		
as a safe practitioner	X			X		
as able to work with other practitioners/health care environment	X	X		X	X	
competent to diagnose and care for the patient	X	X		X	X	X
Statement be used as a standard for self-evaluation						X
Include research related to chiropractic (medicine for WFME)	X	X	X		X	X
Should include social responsibility/service to the community	X	X	X		X	
Committed to life-long learning	X	X		X		
Prepared and ready for post graduate education	X	X				
Include social accountability	X	X				
Educational Standard : Resources	WFME	CCE-Aust	CCE-Can	CCE-Euro	CCE-Int	CCE-USA
Student Teaching Clinic						
Expectation of providing a student teaching clinic	X	X	X	X	X	X
Must be appropriately resourced	X	X	X	X	X	X
Clear and identifiable policies/evidence of						
Patient centred care		X				
Appropriate case mix	X	X	X	X		
Meet all legal requirements			X			
All teaching facilities approved by the “program”		X	X	X		X
Focused on comprehensive and appropriate care	X	X	X	X		
Mechanisms to determine any patient care deficiencies	X	X	X			X
Show proof that the clinics meet the mission / objective statements		X	X	X		X
Provide sufficient supervision	X	X	X	X		X
Evaluate, adapt, improve facilities for clinical training to meet population it serves	X					
Finance						
Adequate & stable finances to support program meet goals/mission	X	X		X	X	X
Must be audited			X			
Fair & equitable refund policy			X			
Length of financial stability		5 years	1 year	5 years	5 years	Long term
Budgetary autonomy	X	X		X		X
Meet legal requirements			X		X	
Budgetary autonomy	X					
Budgetary transparency	X					
Library & Learning Resources						
Adequate support for learning resources to support program goals/objectives	X	X	X	X	X	X
Access to learning resources	X			X	X	X
Adequate for teaching and research	X			X		
A safe learning environment	X					
Information Technology (I.T.)						
Provide I.T. facilities	X	X	X	X		X
Sufficient to deliver the curriculum	X	X	X	X		X
Ensure access to web-based or other electronic media	X					
Effective, ethical, evaluation of appropriate IT and communication technology	X					
I.T. used for independent learning, managing patients, work in health care systems	X					
Physical Facilities						
Provide adequate assets (human & systems) for goals/objectives	X	X	X	X	X	X
Meet legal requirements		X	X	X		
Instructional Aids & Equipment						
Clinic equipment sufficient to meet objectives		X	X	X	X	X
Students obtain acceptable knowledge & skills of standard diagnostic & therapeutic equipment		X		X	X	
Educational Standard : Faculty/Academic staff	WFME	CCE-Aust	CCE-Can	ECC-Euro	CCE-Int	CCE-USA
Appropriately qualified & experienced staff (WFME highly qualified)	X	X	X	X	X	X
Staff should be able to develop, deliver, monitor courses & curricula	X	X		X		X
Stable academic staff		X	X	X	X	X
Balance between chiropractic & non-chiropractic staff (medical/non-medical WFME)	X	X		X		
Balance between F/T and P/T faculty	X			X		X
Minimum chiropractic qualification of 3 years F/T work experience & currently registered	N/A	X Phd	X-Phd	X	X	X
Induction procedures for new staff		X		X		
Staff professional development	X	X	X	X		X
Appropriate administrative staff to support implementation of program	X	X	X	X		X
Regular reviews of staff/management/administrative staff	X	X	X	X		X
Criteria for the balance between teaching/research/service functions	X					
Ensure sufficient knowledge of staff of the total curriculum	X					
Design & implement a staff promotion policy	X		X			X
Take into account staff – student ratios	X	X	X	X	X	X
Educational Standard : Educational Program/Curriculum	WFME	CCE-Aust	CCE-Can	ECC-Euro	CCE-Int	CCE-USA
Curriculum should be consistent with program objectives	X	X		X		
Length of course		10 semesters	4,200 h	300 ECTS		4,200 h
Clinical training length	1/3 total program			1 year		A portion
Number of new Patient encounters for student to graduate		50	35	35		
Number of X-rays studies		60	35			
Number of patient treatments		300	250			
Clinical laboratory tests		25				

There was a diversity of descriptive terms for the intended purpose of the mission/vision/goals/objectives statement. The accreditation standard of the CCE-USA recorded that the intention of the mission statement was to provide for the design of an educational program leading to the qualification of a chiropractor with measureable goals that enable the assessment of the effectiveness for achieving this. The remaining regulatory / licencing bodies intended the mission statement to be used as a measure of the standards to be achieved for a student to graduate. These included becoming a life-long learner, competent, safe, and someone who would serve the community and work with other health professionals.

The CCEs mission statements were more similar than dissimilar when compared to the WFME standards. They were similar in that the CCEs and WFME required that they should be made known to the constituency and community, as well as resulting in the production of a competent practitioner. It was uncommon that CCEs’ mission statements (see Table 3) were found to have included the WFME requirement that the mission statement should result in the production of lifelong learners, be socially accountable, include research, and prepare graduates for post graduate education. It is not unexpected that CCEs would universally include preparation for post graduate education as they are primarily focused on undergraduate training.

#### Domain 2: faculty/staff

Accreditation Standards in all CCEs’ documents stated that staff should be appropriately or adequately qualified and experienced. Three CCEs (Australia, Canada and International) quantified the minimum qualification as being 3 years full-time work experience and current registration for clinical staff. The CCEs of Australasia, Europe and USA expect that the staff should be capable of developing, delivering and monitoring courses and curricula. Two CCEs (Australasia and Canada) required the presence of a least one Ph.D. qualified staff member in the basic sciences.

A wide range of staff scaffolding measures were found in three of the five CCEs (see Table 3). These included induction procedures, professional development and appropriate support from administrative staff. The standards of the CCE-Europe and CCE-USA expected there to be a balance between full-time and part-time faculty though the ratio is not specified. Only the CCEs of Australasia and Europe require that there be a balance between chiropractic and non-chiropractic staff but this ratio is also not stipulated. Finally, all but CCE-Canada demanded the presence of a stable academic staff population.

When compared to the WFME standards the following differences were noted. CCEs used the words “adequate” or “appropriately” qualified staff as compared to “highly” qualified by the WFME. Further, not all CCEs required a balance in staffing levels for the basic sciences and clinical sciences, as well as a balance of full-time versus part-time staff. The WFME standards were unique in that they stipulated the need for a balance between teaching, research and service functions, as well as stating that faculty should have a sufficient knowledge of the total curriculum.

#### Domain 3: resources

##### Teaching clinics

All CCEs accreditation standards matched those of the WFME. All expected the provision of teaching clinics and that they should be appropriately resourced for the delivery of training. All, except the CCE-Europe, required mechanisms to determine if patient care deficiencies existed. All, except the CCE-USA, expected a sufficient case mix of patients. The standards of the CCE-Australasia alone required that care be patient-centred. The notable omission in the CCEs´ standards when contrasted to the WFME standard was the expectation by the WFME that teaching clinics should be evaluated, adapted and improved to meet the needs of the population it serves .

**Table 4:** Curriculum/program subdomains of CCE educational standards

	WFME	CCE-Aust	CCE-Can	CCE-Euro	CCE-Int	CCE-USA
Curriculum Development						
- Faculty must have freedom to design it	X	X		X	X	X
- Freedom to allocate resources necessary to its implementation	X	X	X	X	X	X
- Curriculum committee represented by staff, students, other stakeholders	X	X		X		X
- Modify program in response to feedback from community and society	X	X		X		
Models and instructional methods						
- Curriculum models & instructional methods should be consistent with goals of the institution	X	X	X	X		X
- Curriculum should include multiple learning models/appropriate learning models methods	X	X		X		X
- Students responsible for their learning process	X	X				
- Students should be prepared to be lifelong & self-directed learners	X	X	X	X	X	X
- Should facilitate higher-level learning			X	X		
Structure		X				
- Institution should document the content, extent and sequencing of the courses & how they are integrated into a coherent program	X	X	X	X	X	X
- Basic sciences and clinical subjects should be integrated in the curriculum	X	X	X	X	X	X
- The average student loads should be reasonable		X	X			
Program Content						
- Should ensure achievement of the clinical competencies	X	X	X	X	X	X
*Principles and practice of chiropractic*		X	X			X
- Identify & incorporate a profile of the philosophical concepts & principles of chiropractic	N/A	X			X	X
- The development of chiropractic practice (medical practice)		X			X	X
- This will create an understanding of the position of chiropractic (medicine) in health care system	X	X	X		X	
*Basic sciences*						
- Identify & incorporate those basic sciences that create an understanding of the scientific knowledge, concepts, methods, fundamental to acquiring clinical science	X	X	X	X	X	X
- Should be adapted to the health needs of the society	X	X				
- EB health care must be taught throughout the curriculum	X	X		X		
- Contains a list of basic sciences subjects to be taught	X	X	X	X	X	X
*Clinical sciences*						
- Students must have adequate patient experiences & opportunities to acquire sufficient clinical knowledge, skills & attitudes to assume appropriate clinical responsibility on graduation	X	X	X	X	X	
- List of clinical sciences	X	X	X	X	X	X
- Basic sciences staff and clinicians should collaborate around clinical problems	X			X		
- Contains a list of clinical subjects & skills to be taught	X	X	X	X	X	X
*Behavioural and social sciences and ethics*		X	X	X		
- Identify and incorporate behavioural & social sciences and ethics that enable effective communication, clinical decision making & ethical practice	X	X	X	X		X
- These adapted to scientific developments in chiropractic & changing demographic & cultural contexts & to health needs society	X	X				
- Contains a list of behavioural & social science & ethics subjects to be taught	X	X		X		

##### Finances

All CCE standards documentation contained the expectation that sufficient finances should be available for programs to meet their overall aims. The CCE-Australasia, Europe and USA required programs to have sufficient autonomy or control over their financial resources to achieve their overall objectives. All CCEs required budgetary considerations to encompass the most recently enrolled graduates, except CCE-Canada who required a fiscal policy for a single year. The CCEs of USA and Canada required audits. The CCE International and CCE-Canada expected programs to meet all legal accounting procedures.

The WFME standards were more general in nature but were similar to the varying CCEs´ financial standards with the requirement that programs should have budgetary autonomy and a transparent plan to meet its educational objectives. No timelines for budgetary projections or proof of financial stability going forward were suggested by the WFME standards.

##### Library and learning resources

All accreditation standards required that programs should provide appropriately staffed library and learning resources sufficient to support the educational institutions mission and goals. The documents for two CCEs stated that it should be accessible (CCE-USA & International). Adequate information technology facilities for teaching the curriculum were expected by all CCEs except the CCE-International.

CCE standards generally met those of the WFME. The CCE-USA and the WFME both contained the requirement of the need to provide a safe learning environment for students. Detail as to what constitutes “adequate” or “appropriate” is not provided by WFME or CCE standards.

##### Physical facilities and Instructional aids and equipment

All accreditation standards expected the provision of adequate clinic and learning equipment for achieving mission objectives. The CCEs of Australasia and Europe obliged programs to provide these to a level so that students can obtain acceptable knowledge and skills of standard diagnostic and therapeutic equipment.

The educational standards of all CCEs expected the provision of adequate assets/facilities for the programs to meet their objectives. The standards of CCE-Australasia, CCE-Canada and CCE-Europe expected that these should meet legal and safety requirements.

The standards as recorded by the CCEs´ documents, although worded differently, appeared uniform and comprehensive and were comparable with the WFME standards within this subdomain.

#### Domain 4: educational programs/curriculum

##### The number of contact hours and patient consultations stipulated for training

Although all CCEs related the duration/extent of courses, this was defined in different ways: as five years of study/10 semesters (CCE-International/CCE-Australasia respectively), 4,200 h (CCE-Canada and USA) and 300 European Credits Transfer Scheme (CCE-Europe). The WFME makes no recommendation for an appropriate length of time for medical training.

The length of clinical training for chiropractic students was set at 1 year and a minimum of 35 new patient assessments by the CCE-Europe standards. The same number of new patient assessments was also found in the CCE-Canada accreditation standards, as well as 35 X-ray series and 250 patient treatments. This number was increased to 50 new patients, 60 X-ray series, 300 patient treatments and 25 clinical laboratory tests in the CCE-Australasia standards. The CCE-USA accreditation standard was found to record the length of clinic training as “a portion of the course”. The WFME require that a reasonable part (defined as one third of the program) should be spent in planned contact with patients in relevant clinical settings.

In summary, there appeared to be agreement between CCEs on the total program course length, but differing descriptors were used. However, there was considerable variation between clinical training requirements of CCEs and all of these were different to the WFME standards which contained the stipulation that at least one third of the program to be spent in patient contact.

##### The curriculum

All CCEs and the WFME standards were found to have recorded that the faculty should have the freedom to design the curriculum as well as the resources to implement it (See Table 4). Curriculum development was not specifically mentioned in the CCE-Canada standards. However it is possible that curriculum development could be viewed as a component of the overall assessment of the program under the domain of “Evaluation, Planning and Effectiveness”.

Two of the standards (Australasia and Europe) and the WFME required that the curriculum committee should be represented by staff, students and other stakeholders and that the program should be modified in response to feedback from society.

There was uniformity among all CCEs and WFME standards that the curriculum should prepare students to be lifelong and self-directed learners. All CCEs also dictated that curriculum models and instructional methods should be consistent with the stated goals of the chiropractic program. The requirement that curriculum models should facilitate higher learning was found in the CCE standards of Canada and Europe. Only the Australasian and WFME standards made the demand that students should be responsible for their own learning processes.

All CCEs and WFME accreditation standards for chiropractic institutions were instructed to incorporate and integrate the basic sciences in a coherent manner that creates an understanding of the scientific knowledge, concepts, and methods fundamental to acquiring clinical science knowledge. However, only the CCE documents of Australasia and Canada required that the “average” student loads be “reasonable”. The CCEs of Australasia and Europe and the WFME documents expected evidence-based health care to be taught throughout the curriculum. This topic is covered in more detail in a previous publication [24].

There was wide agreement in all CCE documents that students should be taught adequate clinical, behavioural and social sciences, and ethics, and that they should have access to “experiences” with patients and opportunities to acquire sufficient clinical knowledge, skills, and desirable attitudes to assume appropriate ethical clinical responsibility upon graduation.

Some requirements were only recorded in one CCE accreditation standards: basic science and clinical staff should collaborate around clinical problems (CCE-Europe), and curriculum should be adapted to scientific developments and the health needs of society (CCE-Australasia).

In summary, some CCEs did not contain the following component areas found in the WFME standards; the curriculum committee should be represented by all stakeholders, the curriculum should be modified by stakeholders and should include appropriate learning models that require students to be responsible for the learning processes as well as facilitating life-long and higher learning processes, and average student loads should be reasonable.

##### Program content

All CCE agencies and the WFME standards were in accord that the program content should ensure achievement of the stated clinical competencies(See Table 4). Differences were noted in that the CCEs of Australasia, USA and International did require the inclusion of the philosophical concepts & principles of chiropractic or the development of chiropractic practice, but those of Canada and Europe did not. All CCEs’ accreditation standards were similar in the stipulation that program content should include the sub-domains of basic, behavioural, social and clinical sciences, further, that these subjects should be adapted to the changing demographics, cultural contexts and health needs of society. The WFME, CCE-Australasia and CCE-Europe required that evidence-based health care be taught throughout the curriculum.

##### Subjects required for basic, behavioural and clinical sciences

The CCE accreditation standards of Australasia, Canada, Europe and USA contained lists of subjects required to be taught under the subject areas of principles and practice of chiropractic, basic, clinical and behavioural sciences whereas the CCE-International did not list any required subjects. There were 53 subjects stipulated across these four remaining CCE subject lists (Table 5). Thirteen of these were common to the four CCE standards documents (excluding the CCE-International); anatomy, biochemistry, microbiology, neurology, pathology, physiology, biomechanics, nutrition, orthopaedics, diagnostic imaging, physical, clinical and laboratory diagnosis, adjusting techniques, and spinal analysis. Twenty-three were found in only one CCE educational standards document and included subjects such as practice ethics and management (CCE-Australasia); mental health assessment (CCE-Australasia); reflective practice skills, legal aspects of practice and chiropractic history (CCE-Europe); and wellness, toxicology, extremity adjusting (CCE-USA).

**Table 5:** Subject lists expected to be part of chiropractic program curriculum in CCE educational standards

	WFME	CCE-Aust	CCE-Can	CCE-Euro	CCE-Int	CCE-USA
Fundamental knowledge of health sciences	X				X	
Normal & abnormal patho-physiology of NMSK system	X				X	
*Basic Sciences*						
Anatomy	X	X	X	X		X
Biochemistry	X	X	X	X		X
Biophysics		X		X		
Genetics	X	X		X		
Immunology	X	X		X		
Microbiology	X	X	X	X		X
Neurology	X	X	X	X		X
Molecular & cell biology	X	X	X	X		
Pathology	X	X	X	X		X
Physiology	X	X	X	X		X
Public health	X	X	X	X		
*Clinical sciences*						
Adjustive technique	N/A	X	X	X	X	X
Biostatistics	X		X			
Biopsychosocial model of pain	X			X		
Biomechanics		X	X	X	X	X
Chiropractic history	N/A			X		
Clinical decision making	X					X
Diagnostic imaging procedures		X	X	X	X	
Dermatology	X	X	X			
Epidemiology	X		X			
Ergonomics		X				
Extremity adjusting	N/A					X
First aid & emergency procedures	X	X				X
Geriatrics	X	X	X			
Gynaecology	X	X	X			
Legal aspects of practice	X			X		
Mental health assessment	X	X				
Nutrition / dietetics		X	X	X		X
Obstetrics	X	X	X			
Ophthalmology	X	X				
Oral & written communication skills	X			X		
Organ systems	X					X
Orthopaedics	X	X	X	X	X	X
Otolaryngology	X	X	X			X
Pain management	X			X		
Paediatrics	X	X	X			
Patient management (active & patient centred)	X			X		X
Pharmacology	X	X		X		
Physical, clinical & laboratory diagnosis	X	X	X	X	X	X
Psychology	X		X			
Practice ethics	X	X				
Practice management		X		X		
Principles & practice of chiropractic	N/A		X			
Professional practice ethics & interprofessional collaboration	X		X			
Reflective practice skills				X		
Research methods & procedures	X		X		X	
Rehabilitation & therapeutic modalities	X	X		X	X	X
Sociology	X		X			
Special populations				X		
Spinal analysis	N/A	X	X	X		X
Toxicology						X
Wellness						X

Subjects that were in the WFME lists but not in all CCE lists included genetics, immunology, public health, biostatistics, clinical decision making, dermatology, epidemiology, first aid emergency procedures, geriatrics, gynaecology, legal aspects of practice, mental health assessment, obstetrics, ophthalmology, pain management, pharmacology, psychology, practice ethics, research methods and procedures, and sociology, and evidence-based medicine.

## Discussion

This systematic review is the first to show similarities and differences between accreditation standards as prescribed by CCEs. It also compared the chiropractic standards to those of the WFME. Generally, there were many differences but also some similarities and it is apparent that the WFME standards can be used for further guidance on how to improve and homogenize chiropractic accreditation standards.

### Objective 1: definitions of educational standards

In a previous paper comparing definitions of competency [25] it was noted that one broad definition was not suitable for all professions [26] and that what is required are specific definitions that have sufficient detail and clarity to be professionally and educationally useful [27].

In relation to the five CCEs definition of the term “educational/accreditation standard”, it was actually present only in two instances, and only one of these included detail specific to chiropractic education. Nevertheless, the domains of the accreditation standards were more similar than dissimilar across the five CCEs. The differences, however, became increasingly evident when the detail of the component lists describing the domains were compared.

### Objective 2: domain analysis of educational standards

There were considerably more similarities than differences in the domains of the accreditation standards of the CCEs. One example of a difference is the domain of “Ethics and Integrity” which was listed at the Domain in one CCE and at the subdomain level in 2 others. Concerns have been raised over chiropractic business ethics in the past [28]. These have included unsubstantiated claims in patient brochures [29], anti-immunization views [30] and the sale of “good health” products [31] among others. One solution suggested has been the establishment of a broader based and more congruent undergraduate ethics curriculum [32]. We could no evidence to suggest that by including this as a domain in accreditation standards, although intrinsically appealing, would impact on poor business ethics in future graduates.

It is important that standards follow educational and technological development, so that, for example, they relate to use of the internet in educational institutions. Consequently, we would recommend that all CCEs consider including “Distance/correspondence education” in their accreditation standards so as to embrace all “on-line” teaching.

### Objective 3: selected subdomain analysis of educational standards

#### Domain 1: mission/vision/goals/objectives

One strategic tool that both academics and practitioners have deemed critical to the success of any health-care organization is the development of a meaningful mission statement [33]. The presence of the requirement for a mission statement or objectives in all CCEs and the WFME standards reflects this attitude. There is literature on the difficulties in creating effective mission statements [33, 34], and it is important that CCEs put some effort into the formulation of mission statements with an understanding that they are used with the view of implementation.

There were considerable differences between CCE accreditation standards on who should define the mission statement. This varies from basing it on input from all stakeholders (staff, profession, patient groups and society) through to an obligation to make it known to them. The chiropractic profession is not homogeneous [25]. Programs may find themselves in an environment where there are two professional groups, one being vitalist or “philosophically” driven while the other is based on biological plausibility and, as far as possible, on evidence [35]. It is the authors’ contention, and that of others, that the greatest opportunities for chiropractic lie in the integration into mainstream healthcare and this must be founded on evidence-based health care [36, 37]. To this end mission statements cannot be based on all stakeholders input if they are non-evidence based, such as vitalism or “traditional” chiropractic philosophy. CCEs should be clear in their directives to programs on which input should and should not be given consideration for the construction of their mission statements.

It should be noted that some programs must align their mission statements with that of the university systems they are part of. Due consideration should be given to this imperative as more chiropractic institutions become integrated with government funded universities.

Tabulation of the mission statements revealed that they differed in purpose. Some intended it to be used as a standard for program self-evaluation, whereas others thought it should be used for describing the educational strategy for producing the competent graduate. Thus the graduate was described differently across CCEs standards. Careful consideration should be given to the use of detailed and specific terminology in order to remove descriptive vagueness. By doing this with mission statements they can be better used for their intended purpose of course evaluation and can fully inform educators of the “end product” they are required to produce. We would recommend an increase in the descriptive language specific with respect to their intended purpose for mission statements. Consideration should be given to including identified terms from this analysis such as “lifelong learner”.

#### Domain 2: resources

##### Teaching clinics

There was a consensus among CCE standards on the need for a teaching clinic, but little uniformity on the specific detail for appropriate standards. For example, non-uniformity was demonstrated in that not all CCE standards included the stipulation for patient centred care, meeting all legal requirements, providing comprehensive and appropriate care, or mechanisms to determine if there were any patient care deficiencies present. The amalgamation and adoption of evidence-based standards, such as these, has been shown to enhance the student experience and produce graduates of a higher quality and should be adopted by all CCEs [38].

Our review revealed that all but the CCE-USA mentioned the case-mix of patients. Although none addressed the issue of students having to recruit their own patients, CCE-Canada and CCE-Australasia limited the proportion of family and/or friends allowed. Case mix has been shown to be positively related to learning outcomes, practitioner reported self-confidence, comfort level and learning benefit in medical education [39]. This suggests that chiropractic students being exposed to a broader case mix, such as hospital settings, could enhance their learning and as such should be considered.

##### Finances

Quality international business standards would, at a minimum, require the meeting of all legal requirements, adequate provision of finances to allow all students currently enrolled to graduate, transparent annual reporting, and independent auditing [40]. This minimum standard was not uniformly prescribed across all CCE accreditation standards. For example, there was not a uniform requirement for programs to have autonomy or control over their budgets. The authors recognise that there may be variations between geographic locations where other agencies may regulate or accredit various elements in chiropractic programmes. For example regional agencies may have robust financial evaluation standards which reduces the need for CCEs to perform to the same level.

A particular problem could be that chiropractic programs amalgamated with universities may have a reduced capacity for input or control over their budgets. This should be guarded against by clearly stating this requirement in the CCE accreditation standards.

#### Domain 3: faculty/academic staff

Accreditation standards in all CCEs stated that staff should be appropriately qualified, experienced and supported by appropriate administrative staff. The most commonly expressed qualification was registration and three years practice experience. The authors believe that if chiropractic education is to become a respected member of the health professions then its staff should be encouraged, via educational standard requirements, to attain the broader health industry standard i.e. a research doctorate such as a Ph.D. and that the number of staff with this requirement should be considerable. At a minimum the requirement should be to adopt the WFME standard of “highly qualified” for program staff.

CCE standards did not include the WFME standards for faculty to have a balance between teaching, research and service functions, as well as having a sufficient knowledge of the total curriculum. This would appear to be important. Medical faculty who had out-dated research methodologies, poor skills in critical evaluation of medical information and authoritarian teaching relationships were found to be barriers to the adoption of evidence-based medicine [41]. The major self-report vocational concerns of medical faculty also related to research publications and teaching [42]. It is likely that chiropractic educators would be similar, however no research could be found to verify this.

#### Sub-domain 4: educational program / curriculum

The curriculum / educational program was the largest domain for analysis. Two methods of determining a student’s preparedness for graduation were found. One was the student having attained a level of competence as specified in the graduate competency standards. The second was evidence of having completed a specified number of new and returning patient assessments and treatments. Medical education is moving away from a time or numbers-based system and toward attaining competencies [43]. However, some have suggested that both should be used [44, 45]. We could not find any research which gave direction as to what numbers of patient treatment encounters were optimal for producing competent chiropractic graduates. It is important that educators keep informed of recent developments in this area and that they re-consider the old system of counting numbers of patient visits to justify clinical competency. Research is needed to determine the impact that the number of clinical encounters or the amount of time spent in a training clinic has on students attaining competency to practice safely and effectively. It must be remembered, that when programs are aligned with universities, they have to meet the expectations also of the university.

All CCEs accreditation standards required curriculum models and instructional methods to be consistent with the goals of the institution. Although current evidence suggests that the best way to teach, at least for some subjects [46], is by combining multiple pedagogical resources to complement one another and that students appear to learn more effectively when multimodal and system-based approaches are integrated [40], this was not generally required across the CCEs. Therefore, CCEs need to recommend suitable staff development and upgrading in the pedagogic domain to ensure that not only the contents of the courses but also the delivery of the courses is suitable.

Program content was generally conceptualised in all CCE accreditation standards as consisting of four components. However, there was considerable diversity in the subjects mandated for each of these components among CCEs. It is possible that this was in part due to differing scopes of practice between the CCEs. Medical education has recognized the need to delineate the subjects, and areas within subjects, required for the purpose of graduating the safe and effective medical practitioner. For example studies have been conducted to try to identify which areas of anatomy constitute the required body of anatomy knowledge [47, 48]. No studies could be identified for purposes of graduating the safe and effective chiropractic practitioner. Such studies could create a clearly defined knowledge base from which programs could produce quality graduates. It is expected that this core or common body of knowledge would change over time. For example, it is possible that there will be an increasing emphasis on MRI and ultrasound imaging modalities [49, 50].

#### Methodological considerations

A potential weakness of this study is the subjective nature of the interpretation of the structure for the analysis of the domain and component statements. Our choice of sub-domains may differ from others. There may be other possible constructs for analyses which may impact on the differences and similarities observed.

The strengths of this review are that it did take a systematic approach and that the two investigators extracted the information blindly and with a high level of agreement. Further, all available information was covered and analysed.

## Conclusions

This systematic review investigated and identified similarities and differences between the various CCEs and thereafter with the WFME in their prescribed accreditation standards. The main similarities between CCES were found in relation to the structure and terms describing the domain level of accreditation standards. However, differences were noted in the interpretation of those terms. These differences became more pronounced at the component descriptive level. These included differing intended purposes of the mission statement, standards for faculty staff, requirements for clinical training by students, program budgetary autonomy and transparency, the need for chiropractic philosophy and history, and which subjects should be taught in basic, behavioural and clinical sciences. Consequently, a series of recommendations were made in an attempt to bring parity between CCEs’ educational standards and best medical international practice (Table 6). The adoption of these has the potential to create a homogenised, internationally consistent, and high quality set of accreditation standards.

Differences were also found in relation to the WFME, mainly in relation to the scope of the mission statement, levels of qualifications of faculty, the balance of research, teaching and service for academic staff, and that evidence-based healthcare be taught throughout the curriculum.

Variations in international accreditation standards may be influenced by CCEs differences in enforcement standards. This suggests the need for studies defining similarities and differences of chiropractic program self-evaluation reports and rejoinders to CCE responses, CCE accreditation/inspection team reports, and final reports of findings.

### Recommendations

**Table 6:** Summary table of recommendations

	Recommendations in relation to educational standards	Justifications
Recommendations for definitions of “Educational Standard”		
1.	All CCE documents should contain a definition of the term “educational standard” and it should provide enough profession-specific detail to be professionally useful for chiropractic programs.	Chiropractic educators would better understand the concept of an educational standard if it was detailed and can thus more easily meet the required standards
Recommendations for the domains of Educational Standards		
3.	Add the domain “distance education” to educational standards	Quality of content and assessment of on-line material should be standardised to ensure uniform and high quality standards.
Recommendations for the subdomains of Educational Standards		
4.	Perform a literature review for empirically based methods to successfully formulate and implement a mission statement	Make it easier to prescribe and provide an effective mission statement
5.	Include comprehensive and specific terminology for identifying and explaining the purpose of the mission statement	Educators should have a clearly defined goal in order assist them build a quality program
6.	All appropriate stakeholders should be considered and listened to in the developing of mission statements	Aligns chiropractors with societal needs and expectations
7.	Chiropractic programs mission statements should include a social responsibility.	Also aligns chiropractors with societal needs
8.	The clinical aspect of chiropractic programs should take place partly in hospitals	To provide an appropriate patient case mix exposure for chiropractic students
9.	There should be a minimum set of financial standards in accord with best international business practice	To ensure the long term survival of the course and protection of students and staff.
10.	Chiropractic program staff must include people with PhD degrees.	To improve the educational standing of chiropractic education.
11.	CCEs should encourage research to inform educators of the optimal number of patient numbers, hours or competencies required for student training	To increase the likelihood that graduates achieve the highest levels of competence
12.	There should be a requirement for multimodal learning in curricula	To improve students’ learning outcomes
13.	CCEs should encourage research into which types of learning work best for specific subjects for chiropractic students	To maximize the teaching/learning situation as much as possible
14.	CCEs should help identify the “core” material required for chiropractic graduates	To economize time at its maximum and keep updated on scientific changes and developments in clinical practice

## Abbreviations

CCE: Council on Chiropractic Education
USA: United States of America
WFME: World Federation for Medical Education
